# Initiation of oral hepatitis C virus treatment: Which barriers are pertinent for ambulatory individuals with a history of illicit substance use? A qualitative interview study

**DOI:** 10.1002/hsr2.1814

**Published:** 2024-01-22

**Authors:** Selina Barbati, Johannes Strasser, Samuel S. Allemann, Isabelle Arnet

**Affiliations:** ^1^ Pharmaceutical Care Research Group, Department of Pharmaceutical Sciences University of Basel Basel Switzerland; ^2^ University Psychiatric Clinics Basel Switzerland

**Keywords:** barriers, direct‐acting antivirals, hepatitis C, people who inject drugs, qualitative research

## Abstract

**Background and Aims:**

The World Health Organization has set a goal to eradicate hepatitis C virus (HCV) by the year 2030. Nonadherence to HCV treatment has substantial economic implications due to high treatment costs, among others. Barriers to start HCV treatment may be critical. The aim of this study was to assess pertinent barriers to HCV treatment in ambulatory patients with a history of illicit substance use and to compare them to the literature.

**Methods:**

Barriers to HCV treatment mentioned by the key risk group (i.e., people who inject drugs) were retrieved from literature through a pragmatic literature search. From 34 published articles, we identified 80 modifiable barriers that were bundled in 23 items within the four topics “Personal difficulties and barriers to treatment,” “Personal motivation to be treated,” “Knowledge about the disease,” and “Received information about the medicine.” In‐depth semistructured interviews were performed face‐to‐face with ambulatory patients from the University Psychiatric Clinics in Basel, Switzerland. Transcripts were coded inductively.

**Results:**

Interviews were performed with seven individuals (mean age: 48.3 years; range: 38–63 years; one woman) treated with oral direct‐acting antivirals between 2014 and 2022. Thirteen barriers to start HCV treatment were mentioned that corresponded to the five categories: information, attitudes, swallowing difficulties, social environment, and unfavorable lifestyle. The barrier “swallowing difficulties” emerged exclusively from the statements provided by the interviewees.

**Conclusion:**

Barriers to the initiation of HCV treatment indicated by our interviewees clearly differed from the literature. Notably, the challenge of swallowing medicines may be particularly relevant for physicians prescribing and pharmacists dispensing HCV medication.

## 1 INTRODUCTION

Hepatitis C is a virus‐related inflammation of the liver caused by infection with the hepatitis C virus (HCV). HCV is mainly transmitted through direct percutaneous exposure to blood such as blood transfusions, parenteral drug administrations, or injected drug abuse.^1^, ^2^ Approximately 80% of HCV transmissions are related to injecting drug use.^3^ Chronic progression of hepatitis C develops in 75%–80% of HCV‐infected individuals^4^, ^5^ and is associated with severe secondary diseases (e.g., end‐stage liver disease, hepatocellular carcinoma, liver‐related death) and increased mortality.^6^ The key risk group for acquiring HCV are people who inject drugs (PWIDs). Among PWIDs, HCV is one of the most common chronic diseases and represents a major public health burden.^7^

In 2016, the World Health Organization (WHO) set a target to eradicate hepatitis B and C as major public health threats by the year 2030. Globally, 58 million people are living with chronic HCV infection, and only in 2019, there were 1.5 million newly infected people. In 2019, about 290,000 people died as a consequence of HCV‐related infections.^8^ In Switzerland, approximately 32,000 individuals live with HCV^9^ while the proportion of statistically recorded illicit substance users infected with HCV varies between 7700 and 15,400.^9^ Every year, around 500 individuals who inject illicit drugs are diagnosed with HCV infection.^10^ The prevalence is estimated at 0.7% among the general population, not including the risk group, and 45.8% among high‐risk individuals.^11^

The current standard of care in HCV‐infected individuals consists of oral administration of direct‐acting antivirals (DAAs). In most patients, these drugs are well tolerated and highly efficacious, with a cure rate of over 95% after a short treatment duration of 8 or 12 weeks.^12^ Successful HCV treatment prevents the occurrence of secondary diseases and the possibility of HCV transmission, which can ultimately reduce health care costs.^13^ Given the low‐risk—high‐benefit profile, the successful eradication, and the high treatment costs, identifying potential barriers to treatment initiation seems crucial. However, multiple barriers to treatment are still present at the level of patients, providers, and systems.^14^ Patient‐level barriers include, for example, poor vein health or experienced stigmatization.^15^, ^16^ Significant barriers at a provider level include lack of required knowledge among practitioners and perceived patient nonadherence.^15^ Finally, at the system level, limited access to health care services and restrictive reimbursement remain key barriers to HCV treatment.^15^ Addressing modifiable barriers is crucial because only these barriers can be actively targeted and overcome through the provision of interventions.^17^

There are only a few qualitative studies describing barriers to DAA treatment from a patient's perspective among the PWID population.^15^, ^18^ Examples are low priority or finding the right “time” for treatment impeded patients to seek HCV treatment.

Previous research concentrated mainly on the health care provider's perspective of what barriers the patient faces,^19^, ^20^, ^21^ and less on a patient's perspective.^16^, ^22^, ^23^

To address this research gap from the patient's perspective, we aimed to assess pertinent barriers to DAA treatment in ambulatory patients with a history of illicit substance use in Basel, Switzerland, and to compare them to published barriers.

## 2 MATERIALS AND METHODS

### 2.1 Compilation of barriers

A single literature search was performed in March 2021 in the MedLine database through PubMed with the combination of keywords and their synonyms: barriers, facilitators, HCV, treatment, initiation, PWID, opioid agonist treatment (OAT), and patient statement. Upon review of article titles and abstracts, 34 articles addressing barriers to HCV treatment published within the timeframe 2007 and 2020 were considered. We identified 203 barriers at the level of patients, providers, and systems. After eliminating duplicates and merging synonym terms, we obtained 80 modifiable barriers on a patient's level that were summarized into 23 items (Table 1, items in bold). Four blocks were defined according to their topic: (i) Personal difficulties and barriers to treatment; (ii) Personal motivation for treatment; (iii) Knowledge including self‐assessment about the disease and its treatment; and (iv) Received information about the medicine. Similarities of content were merged to generate harmonized items. Completeness of topics was checked with the Patients' Lived Experience with Medicines framework.^25^ Because DAAs are administered orally and represent a revolution in HCV treatment compared to former injections,^26^ two items not described in the literature were added concerning the duration of the oral treatment and the number of tablets taken per day.

**Table 1:** Final 30 items according to the topic that were used to conduct the interviews after transformation into questions.

Topic	Number	Item
Personal difficulties and barriers to treatment	1	**Major difficulties or barriers to hepatitis C treatment**
	2	**Difficulties in initiating hepatitis C treatment**
	3	**Integration of medication intake into daily life**
	4	**Difficulties to persist with hepatitis C treatment**
	5	**Understanding information on hepatitis C medicine and its treatment**
	6	**Trust in the prescribing physician**
	7	Preference of two treatment regimens (three tablets at a time, once daily for 8 weeks, or one tablet once daily for 12 weeks)
	8	**Support during hepatitis C treatment**
	9	**Suggestions on how to reduce or overcome the difficulties or barriers**
Personal motivation for treatment	10	**Motivation to undergo hepatitis C treatment**
	11	**Reasons for being motivated or not motivated to undergo hepatitis C treatment**
	12	**The way the diagnosis was received**
	13	**Importance of being treated with hepatitis C medicines**
Knowledge about the disease and its treatment	14	Self‐evaluation of the knowledge about hepatitis C disease and its treatment
	15	Given reasons for the rating of the self‐evaluation
	16	Source of obtained knowledge
	17	**Knowledge about the health consequences of a hepatitis C infection**
	18	**Knowledge about the onset of the health consequences over time**
	19	**Knowledge about the ideal starting moment for hepatitis C treatment**
	20	**Knowledge about the treatment duration of the hepatitis C treatment**
	21	**Knowledge about the need for treatment duration**
	22	**Knowledge about re‐infection with hepatitis C virus after successful treatment**
	23	**Knowledge about the infection of other people after successful treatment**
	24	**Knowledge about the vaccination against hepatitis C virus**
	25	**Knowledge about the vaccination against hepatitis A and B virus**
Received information about the medicine	26*	What did your doctor tell you the medication is for?
	27	**Fill/refill method for the hepatitis C medication**
	28	**Information obtained from the health care provider for what the medication was prescribed**
	29*	What did your doctor tell you to expect?
	30*	How did your doctor tell you to take the medication?

*Note*: The 23 items extracted from the literature are marked in bold, and the IHS^24^ prime questions are marked with an asterisk (*). An unauthorized English version following a single forward translation from German was conducted by S. B.

Abbreviation: IHS, Indian Health Service.

### 2.2 Development of the interview guide

Two investigators (S. B., I. A.) transformed the items into questions and utilized cognitive debriefing to verify their comprehensiveness and understanding. Additionally, the three prime questions from the Indian Health Service^24^ were added as validated conversation openers. We emailed the interview questions to 25 people in the acquaintance of the research group for evaluation. They were asked to assess factors including comprehensibility, logical sequence of the blocks, clarity and logical structure of the interview, and intrusiveness. Answering options ranged from 1 to 4 (1 = *does not apply*, 2 = *does rather not apply*, 3 = *rather applies*, 4 = *applies*). Answers to negatively formulated questions were reverse‐coded so that a higher score indicates a positive evaluation. A free comment was possible after each section. Out of the 25 participants, 11 evaluated the interview questions (mean age: 44.2 ± 13.6 years; range: 26–68 years; one bachelor's student and four PhD students with a master's degree; one master's graduate; three PhDs; two professors; four women). Comprehensibility was rated with an average value >3.0 for each block. The logical sequence of the blocks was rated with an average of 2.9, the intrusiveness of the items with 3.7, and the clarity and structure of the interview with 3.1. In total, 122 comments were provided, primarily related to item sequence, wording, and sentence structure. A total of 88 comments (73%) were accepted. The rejected comments were of general nature or concerned the phrasing of the items. One comment on facilitators was accepted and added as an additional item (i.e., what helped the person to overcome the barriers). The revised interview questions underwent face‐to‐face testing with an advanced practice nurse specialized in psychiatry who embraced the role of a patient. The testing resulted in three minor and two major practical adaptations, including providing a hard copy of the interview questions at the beginning of the interview and specifying the interview duration. All comments were implemented.

The final interview guide consisted of 30 questions (Table 1), grouped into four topics: Personal difficulties and barriers to treatment (nine questions), Personal motivation for treatment (four questions), Knowledge about the disease and its treatment (12 items), and Received information about the medicine (five questions; Table 1). The answer formats were open‐ended (nine items), close‐ended (19 items), or Numeric Rating Scale from 0 to 10 (two items). One numeric scale question was to self‐evaluate the general level of knowledge about HCV (1 = *I do not know* to 10 = *I am an expert*). A higher score indicated greater knowledge. Although knowledge is considered a major determinant of disease management in general and of adherence to medication in particular,^27^ its assessment is complex. Typically, knowledge questionnaires focused on disease‐related knowledge, aligning with current recommendations, especially concerning pharmacological treatment and remission strategies.^28^ These questions targeted specific areas such as medication names, with respondents selecting a single correct response.^29^ In the case of statements, the multiple‐choice answer options were True, False, or Don't know.^28^

### 2.3 Study design

This was a qualitative study based on in‐depth semistructured interviews with prespecified analysis.^30^ We recruited individuals from an ambulatory setting (ADS, Ambulanter Dienst Sucht) of the University Psychiatric Clinics in Basel, Switzerland. The ADS is specialized in the treatment of substance use disorders and is dedicated to individuals with additional psychological and somatic disorders and social impairments. Physicians and psychotherapists working at the ADS selectively recruited eligible patients. They excluded patients who were, in their opinion, unlikely to adhere to the study schedule or were unsuitable for any other reason. After the patients were asked to participate in the study, the patient information sheet was handed out, and the research aims were explained. Patients confirmed participation at their convenience, for example, during the next medical visit. No relationship was established before the study commencement between the researcher and the participants. The interviews were performed face‐to‐face in a quiet private space in the ADS facilities by the investigator (S. B.) who was a female licensed pharmacist with 5 years of community pharmacy experience. No repeated interviews were carried out, and at the time of the interviews, no nonparticipants were present. The interviews were audio‐recorded and field notes were taken during the interview. Participants were remunerated with CHF 75. Recruitment of study participants started on July 15, 2021 and ended on January 21, 2022 when data saturation was obtained. The interviewer (S. B.) was free to formulate the questions, change their order, and skip the ones that had already been answered by the participants. After each interview, two investigators (S. B., I. A.) re‐evaluated the interview procedure and adjusted the flow of the questions to optimize the interview quality.

The study was approved by the Ethics Committee of Northwest/Central Switzerland (BASEC No. 2021‐00696). All participants provided written informed consent before the interview. We adhered to the guidelines of reporting observational studies STROBE (Strengthening the Reporting of Observational Studies in Epidemiology).^31^

### 2.4 Study population

The inclusion criteria were: to attend regularly the visits at the ADS; to be 18 years of age or older; to be currently treated or to have been treated with a DAA including a single agent or a combination of several agents (sofosbuvir, ledipasvir, velpatasvir, grazoprevir, elbasvirum, pibrentasvir, voxilaprevir, marketed as Sovaldi®, Harvoni®, Epclusa®, Zepatier®, Maviret®, or Vosevi®); to understand and speak Swiss German or German; to accept the audio‐recorded interview; and to sign the informed consent form. The exclusion criteria were: if, in the opinion of the investigator, there was a likelihood that individuals would not be able to adhere to the study plan or were unsuitable for other reasons; if individuals were unable or unwilling to sign the consent form.

### 2.5 Data analysis

The interviews were transcribed verbatim by one researcher (S. B.) following published transcription rules.^32^ Names and places mentioned by the interviewees were anonymized. A second researcher (A. H.) checked the transcripts. Disagreements were solved in a discussion. The transcripts were not returned to participants for comments and/or corrections.

Transcripts were coded inductively. We used thematic analysis^33^ to summarize the codes and create categories with illustrative situations. Storage and analysis of the data were performed with the Padlet software. An independent researcher (F. D.) who was not involved in the interviews revised the codes, the code structure, and the coded quotes to ensure appropriate and consistent coding. Divergent opinions were resolved by consensus. The findings were not checked with participants to provide feedback. Quotes in English were translated by S. B. from (Swiss) German. We analyzed knowledge scales descriptively and yes/no items and reported the means and standard deviations (SD). No statistical tests were used to analyze the data. We used Microsoft Excel® 2016.

## 3 RESULTS

### 3.1 Interviewees' characteristics

Nine individuals were invited to participate, and all were accepted. Two participants withdrew before the start of the interviews because of health problems and unwillingness to be face to face with an unknown person during the pandemic. In total, seven participants (one woman) participated in the study (Table 2). The interviews lasted on average 30.4 (7.7) min (range: 20.3–44.2 min). All participants finished the interview (no drop‐out). A DAA treatment was initiated in 2014 for one participant and between 2017 and 2020 for five participants, among which one stopped interferon therapy before 2014. One participant received a second HCV treatment and was at the time of the interview still on treatment. All participants who had completed DAA treatment had no active HCV infection at the time of the interview. At the moment of the interview, six participants were involved in a program with OAT, and five with slow‐release oral morphine. All but one were smokers, and all had concomitant use of other legal or illegal substances such as cannabis.

**Table 2:** Demographic parameters of the participants (*N* = 7).

Parameter	Value
Age in years, mean (SD)	48.4 (7.6)
Male, *n* (%)	6 (85.7)
Independent living, *n* (%)	4 (57.2)
Assisted living, *n* (%)	3 (42.8)
HCV medication, *n* (%)	
Maviret® (glecaprevir/pibrentasvir)	3 (42.9)
Epclusa® (sofosbuvir/velpatasvi)	3 (42.9)
Sovaldi® (sofosbuvir) and Daklinza® (daclasavir)	1 (14.2)
Concomitant use of substances, *n* (%)	6 (85.7)

Abbreviations: HCV, hepatitis C virus; SD, standard deviation.

### 3.2 Knowledge about HCV disease and its treatment and treatment preferences

Participants self‐assessed their general knowledge about HCV and its treatment as reasonable, with a mean score of 5.8 (2.0). The specific level of knowledge was rated with mean scores of 5.1 (1.9) regarding HCV disease and a mean of 6.4 (1.9) regarding HCV treatment. Knowledge questions on health consequences of hepatitis C infection over time were answered correctly by all (7/7 or 100%) of the participants, but only two (2/7 or 28.5%) were sure about the onset of the disease. All participants answered correctly on the best starting time and duration of HCV treatment, and all participants were aware of the possibility of re‐infection with HCV after successful treatment completion. Out of the participants, six (6/7 or 85.7%) answered correctly on the infection of other people after successful treatment completion, and they were aware that a hepatitis A and B vaccination existed and not a vaccination against HCV.

Four individuals (4/7 or 57%) preferred an 8‐week DAA treatment regimen with three tablets per day, while three (3/7 or 43%) individuals preferred a 12‐week regimen with one tablet per day.

### 3.3 Barriers to oral HCV treatment initiation

The interviewees expressed multiple difficulties to start a new treatment against HCV, which were summarized into 13 different barriers (Table 3) and grouped into five categories.

**Table 3:** List of patient‐related barriers to oral HCV treatment initiation grouped into five categories with corresponding 13 concrete situations.

Category	Barrier
Lack of information regarding	New treatment options
	Side effects
	Diagnostic procedures
	Reimbursement
Negative attitude towards medication and health care system	False expectations on treatment
	Doubts about the success of the treatment
	Lack of priority of treatment
	Lack of motivation
	Lack of trust in health care providers (physician, pharmacist, institution)
Swallowing difficulties	Swallowing difficulties
Insufficient social environment	Lack of social support (from institution, family, friends)
	Fear of stigmatization
Unfavorable lifestyle	Alcohol consumption
	Concomitant use of legal or illegal substances

Abbreviation: HCV, hepatitis C virus.

The first category includes the most frequently mentioned barriers, which were a lack of information regarding the new treatment options, side effects of the treatment, diagnostic procedures, and reimbursement. Importantly, participants thought wrongly that diagnosis is obtained with invasive methods such as liver biopsy, which contributed to not starting HCV treatment: “Biopsy was the main barrier why I didn't start the treatment” (interview 5, man, 50 years).

The second category includes negative attitudes towards medication and the health care system. Due to the asymptomatic nature of HCV, some participants did not see any urgency for starting the treatment and were therefore not motivated to be treated: “I had the disease and always tried to push it away … I asked myself, why should I get treatment if I don't have any symptoms” (interview 2, man, 53 years). One participant stopped interferon therapy due to the lack of priority of the HCV treatment over substance use: “I actually wanted to stop (intravenous drug use) … and then the stuff with the syringe came again … and then I started injecting drugs again in parallel with HCV treatment” (interview 4, man, 49 years). A negative experience reported by peers contributed to mistrust regarding HCV treatment. One participant reported a dissuasive statement from a provider to start HCV treatment because of the concomitant use of substances: “You have an addiction problem and you will get re‐infected again … let's wait for two years until a new HCV medicine is authorized” (interview 6, man, 45 years). Swallowing difficulty builds the third category. In the fourth category, insufficient social environment posed a major burden. Stigmatization was reported: “I knew when I was infected and I would have liked to have the medication, but they [the hospital] said we don't make any more appointments … it has to do with the social status of opiate addicts or addicts” (interview 6, man, 45 years). An inappropriate lifestyle builds the fifth category. Concerns about the quantity of alcohol allowed to consume during treatment hindered to start treatment: “I abstained from hard alcohol and from immense amounts of beer … but I drink a beer or two a day and this is something which keeps me concerned during the therapy” (interview 1, man, 38 years).

### 3.4 Difference with published barriers

From the 23 (100%) items extracted from published literature, 22 (22/23 or 95%) were mentioned in the interviews. Swallowing difficulties were mentioned by the interviewees but not in the published literature.

## 4 DISCUSSION

To our knowledge, this is the first study that identified barriers to HCV treatment initiation that were pertinent for the mean risk group, that is, patients with a history of illicit substance use who are undergoing opioid agonist treatment in an ambulatory setting.

Our findings revealed 13 barriers to oral HCV treatment initiation. All except difficulty swallowing medicines were mentioned multiple times in the literature.^34^ However, many barriers were not even insinuated by our interviewees, such as limited infrastructure for HCV treatment, long waiting times to get a medical appointment, lack of follow‐up or nonattendance to medical appointments, food insecurity (e.g., side effects on an empty stomach),^35^ or poor patient–provider communication and relationship.^21^, ^36^ These barriers are at the provider's and system's levels. They were not reported by our interviewees, probably because of the specificity of the Swiss health care system. As an example, throughout Switzerland, there are easily accessible specialized addiction treatment centers offering services such as medical care, counselling, or social support.^37^, ^38^ Also, HCV treatment is reimbursed by the health insurance regardless of liver fibrosis stage.^39^ A further explanation for the lack of provider and system‐level barriers might be that the coding of the interviews focused on patient‐centeredness. As an example, “lack of trust in health care providers” was categorized in “Attitudes,” while some frameworks have a shared conceptual view of trust between the clinician, institution, and patient.^40^ Nevertheless, this situation might also be specific to Switzerland, where almost 80% of people with opioid use disorder are enrolled in a program with OAT, that is, are under medical control. Of note, this represents one of the highest levels of coverage worldwide.^41^ Our results show the importance of educating patients on HCV disease and its treatment because the decision not to start treatment can be based on incorrect information. For example, most of the participants were not aware of new short, noninvasive treatment options. Education and knowledge optimization might be successful in encouraging patients to start treatment and enhance adherence to treatment.^34^, ^42^ The possibility of excluding patients from HCV treatment or delaying treatment initiation due to illicit substance use was mentioned by the participants and was also found in the literature.^19^ Thus, it appears that a lack of knowledge on the prescriber's part can also be a barrier to starting a DAA treatment. In any case, the dispense of recent and accurate information is a simple remedy to this situation.

We identified one additional barrier not described in the literature, that is, the difficulty swallowing medicines. To start treatment and reach optimal adherence to medication and ultimately therapeutic success, the ability to swallow medication is essential in all adherence phases (i.e., initiation, implementation, and discontinuation). Although the regimen of oral HCV treatment is short (8 or 12 weeks), difficulty in swallowing pills might represent a fundamental obstacle and incite individuals to skip doses or stop the treatment prematurely. However, the number of daily DAA tablets to ingest differs, with three or one tablet per day, which might be an important element of the decision when a DAA is prescribed. Thus, practitioners should consider the individuals' preferences before prescribing a DAA. In addition, HCV‐infected patients have often several comorbid conditions and thus concomitant medication (polypharmacy). Guzman Ramos et al.^43^ observed that individuals with HCV presented decreased adherence rates to concomitant medication, and a tendency to focus more on short and simple treatment rather than on their concomitant medication. Even if the focus is on HCV, the prescription of a DAA should be placed in light of the polypharmacy and the pill burden should be mentioned to the patient.

We believe that the observed barriers cover the most frequent barriers in the setting of opioid use disorder. Nevertheless, they might be useful for people starting HCV treatment independently of opioid use disorder such as in men who entertain sexual relationships with other men. In that case, the lifestyle barriers (i.e., concomitant use of legal or illegal substances) might be adapted to the current patient situation.

We observed that negative lived experiences, so‐called “horror stories” and wrong beliefs about HCV treatment from the interferon therapy era are still in the minds of the patients.^44^ Incorrect information regarding invasive diagnosis methods of HCV, together with the belief of numerous side effects of the treatment, hindered patients to visit the physician and start HCV treatment. It is well known that myths linked to medicines are persistent,^45^ that many people believe misinformation,^46^ and that debunking and overcoming misconceptions is a complex undertaking.^47^ At the time of the interview, the participants answered the majority of knowledge questions correctly, indicating that PWID individuals were well informed about HCV disease and its treatment at the ADS clinic. However, suboptimal knowledge such as unawareness about DAA treatment hindered some participants from seeking treatment. This is also in line with the literature.^48^ Thus, given the WHO target plan for 2030, it seems appropriate for health care providers to repeatedly ask patients about their specific knowledge and correct it in case of misinformation.

A central lifestyle factor was mentioned as a barrier to starting HCV treatment, that is, ongoing daily alcohol consumption, and concomitant use of legal or illegal substances. There are still many misperceptions that need to be addressed, such as concerns about the compatibility of HCV medicines with daily alcohol consumption. Studies have confirmed that alcohol consumption or intake of legal or illegal substances increases the likelihood of noninitiation of HCV treatment.^34^, ^49^

Several studies^38^, ^50^ have shown a sustained virological response despite missed doses of DAA, suggesting some tolerance for nonadherence, that is, a certain forgiveness of the medicine.^38^, ^51^, ^52^ Yet, the optimal threshold for nonadherence is unknown for oral HCV therapy. Nevertheless, it seems ethically correct before treatment initiation to properly educate and adequately inform patients about the importance of regular and sustained medication intake to achieve treatment success, which is viral suppression. In addition, high adherence to treatment with daily intakes reduces the risk of viral resistance.^53^ In this sense, pharmacies play an essential role in promoting adherence support, especially in cases when it comes to dispensing daily doses for specific situations such as patients receiving opioid substitution treatment. Adherence support should be offered to all HCV patients and pharmacy staff can have a great impact on medication adherence.^54^ This phenomenon was also confirmed in our interviews.

Since January 1, 2022, prescription of DAA treatment is not restricted anymore to infectious disease specialists, gastroenterologists, and addiction specialists with experience in HCV treatment. The Swiss Federal Office of Public Health has abrogated the restrictions, so that every physician is qualified to prescribe DAAs in Switzerland, and costs are reimbursed by health insurance.^39^ This will facilitate future HCV treatment uptake and render the recognition of barriers before treatment even more important. Thus, our compilation of pertinent barriers could serve as a helpful resource in the field of medicine and pharmacy practice for identifying and overcoming potential obstacles in Switzerland or settings resembling Switzerland.

### 4.1 Study strengths and limitations

Our study had several strengths. First, we used in‐depth semistructured interviews. They are flexible and allow for a dialog during the interview.^55^, ^56^ Since HCV is still very stigmatized by society, we claim that individual in‐depth interviews were the better choice compared to focus group discussions that are conducted with several participants together. With individual interviews, the participants' answers tend to be detailed, spontaneous, individual, and vivid. Thus, personal feelings and stories can be voiced, which allows new concepts or themes to emerge.^55^, ^57^ Second, we interviewed seven individuals within the same setting. Although in qualitative research, thematic saturation is generally reached with 11–14 participants in homogeneous groups in the context of inductive thematic analysis, it is assumed that 80% of the themes can be captured with six interviewees.^58^ Because of our highly homogeneous sample and the use of items extracted from the literature, we assume that seven interviews might have been sufficient to reach data saturation^59^ Third, we interviewed patients who had finished treatment with DAAs, although we aimed to assess barriers before treatment initiation. There may have appeared more barriers for patients who did not receive DAA treatment and were not interviewed. Although this might appear contradictory, the a posteriori method is best to collect lived experiences because it eliminates false expectations or guesswork that might not be fulfilled.^60^ We acknowledge some limitations. First, all participants but one were male. This might be due to the gender distribution of HCV patients in the ADS facility; however, no gender‐specific barrier could be identified. Second, given the small sample size and the specific setting, sample bias cannot be ruled out. An analysis with more volunteers is needed to draw generalizable conclusions on this issue. Third, we focused only on PWIDs. Other populations are at risk of acquiring HCV, such as recipients of blood transfusions before 1992, men who entertain sexual relationships with other men, prisoners, or refugees,^61^ but PWIDs represent a key risk group for acquiring HCV. Thus, we claim that the observed barriers are usable for the majority of HCV‐infected people. Fourth, six of the seven interviewees had ended their HCV treatments several years ago. Some participants could not remember every detail of their past treatment. Thus, recall bias and missed answers cannot be excluded. Nevertheless, we assume that major barriers were identified, especially because of the importance of HCV treatment in the observed population. Fifth, the level of knowledge must be interpreted with caution. Because interviewees had already agreed to HCV treatment, they had probably already obtained more information about the treatment and its disease. Sixth, we did not collect data on comorbid conditions that may influence treatment initiation and adherence to treatment. Nevertheless, most questions were in open‐ended format, and if co‐medication had been a barrier, interviewees could have mentioned it. Seventh, we conducted the literature search in PubMed only. A broader search involving additional databases would probably have retrieved a larger number of articles. Nevertheless, we doubt to have detected additional barriers to HCV treatment since the articles retrieved covered a wide range of barriers at the level of patients, providers, and systems. Finally, we used deductive coding for the interviews and free wording for the categories. We cannot exclude that other researchers would have chosen different categories. However, we avoided scientific jargon and stuck to the patients' wording as much as possible. Thus, we claim that our checklist can be used with PWID patients without further language adaption.

## 5 CONCLUSION

This study offers valuable insights into barriers faced by PWID individuals when initiating oral HCV treatment. The identified 13 barriers can serve as a guide for health care providers, including community pharmacists, to determine the pertinent barriers for the individual patient. Notably, the challenge of swallowing medicines may be particularly relevant for physicians prescribing and pharmacists dispensing HCV medication. The usability of our findings can merge into targeted interventions addressing individual barriers in daily practice.

## AUTHOR CONTRIBUTIONS

**Selina Barbati**: Conceptualization; data curation; formal analysis; investigation; methodology; project administration; visualization; writing—original draft. **Johannes Strasser**: Project administration; resources; writing—review and editing. **Samuel S. Allemann**: Funding acquisition; resources; writing—review and editing. **Isabelle Arnet**: Funding acquisition; project administration; supervision; validation; writing—review and editing. All authors have read and approved the final version of the manuscript and have contributed equally to this work.

## CONFLICT OF INTEREST STATEMENT

The authors declare no conflict of interest.

## TRANSPARENCY STATEMENT

The lead author Selina Barbati affirms that this manuscript is an honest, accurate, and transparent account of the study being reported; that no important aspects of the study have been omitted; and that any discrepancies from the study as planned (and, if relevant, registered) have been explained.

## Data Availability

The data that support the findings of this study are available on request from the corresponding author.
